# Magnesium and the Hallmarks of Aging

**DOI:** 10.3390/nu16040496

**Published:** 2024-02-09

**Authors:** Ligia J. Dominguez, Nicola Veronese, Mario Barbagallo

**Affiliations:** 1 School of Medicine, “Kore” University of Enna, 94100 Enna, Italy; ligia.dominguez@unikore.it; 2 Geriatric Unit, Department of Medicine, University of Palermo, 90127 Palermo, Italy; nicola.veronese@unipa.it

**Keywords:** magnesium, aging, hallmarks of aging, healthy aging, frailty, multimorbidity

## Abstract

Magnesium is an essential ion in the human body that regulates numerous physiological and pathological processes. Magnesium deficiency is very common in old age. Age-related chronic diseases and the aging process itself are frequently associated with low-grade chronic inflammation, called ‘inflammaging’. Because chronic magnesium insufficiency has been linked to excessive generation of inflammatory markers and free radicals, inducing a chronic inflammatory state, we formerly hypothesized that magnesium inadequacy may be considered among the intermediaries helping us explain the link between inflammaging and aging-associated diseases. We show in this review evidence of the relationship of magnesium with all the hallmarks of aging (genomic instability, telomere attrition, epigenetic alterations, loss of proteostasis, deregulated nutrient sensing, mitochondrial dysfunction, cellular senescence, stem cell exhaustion, altered intercellular communication, disabled autophagy, dysbiosis, and chronic inflammation), which may positively affect the human healthspan. It is feasible to hypothesize that maintaining an optimal balance of magnesium during one’s life course may turn out to be a safe and economical strategy contributing to the promotion of healthy aging. Future well-designed studies are necessary to further explore this hypothesis.

## 1. Introduction

One of the most remarkable phenomena of recent times is the continuous aging of the population, which has been present for the last century and a half with consequences in all aspects of society [1]. This trend is expected to continue, with an increase of 4.4 years on average by 2040 in 195 nations [2]. Aging is frequently accompanied by a progressive loss of physiological integrity, which renders the person more susceptible to chronic diseases and related disability [3,4], overshadowing this accomplishment of humanity.

Magnesium is a fundamental mineral, indispensable for numerous cellular processes including all oxidative phosphorylation processes, over 600 enzymatic reactions, energy generation, nucleic acids synthesis and stability, protein synthesis, and carbohydrate metabolism [5,6,7,8]. An adequate magnesium status is essential for organs and systems in the human body. Magnesium deficiency is common in late life and has been associated with various age-related chronic diseases [8].

In 2013, nine hallmarks of aging were proposed by Lopez-Otin et al., comprising genomic instability, telomere attrition, epigenetic alterations, mitochondrial dysfunction, loss of proteostasis, deregulated nutrient sensing, cellular senescence, stem cell exhaustion, and altered intercellular communication [9]. Research on the biology of aging has notably progressed based on this proposal. Indeed, new hallmarks of aging have been added to the original ones, including autophagy, microbiome disturbance, and inflammation, among other emerging ones [10,11]. Advances in understanding the mechanisms underlying the aging process and the possible modifiable determinants can reveal insights on how to achieve the healthiest possible aging.

The present narrative review aims to provide an overview of the links between magnesium and the hallmarks of aging as pathways helping us to explain the effects of magnesium on the aging process and age-related chronic diseases.

## 2. Cellular Magnesium Homeostasis

**Figure 1:**
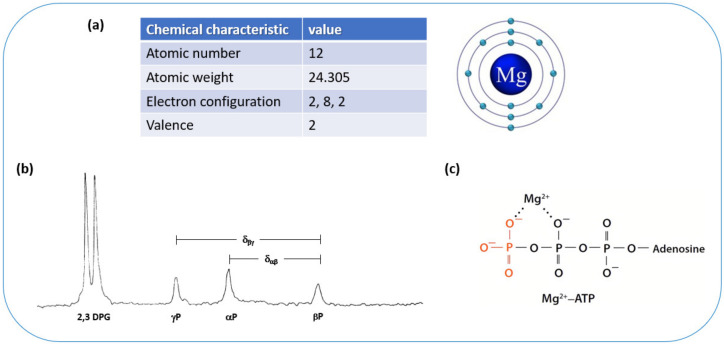
Chemical structure of the magnesium ion and of MgATP. (**a**) Chemical characteristics of magnesium; (**b**) the Fourier transform ^31^P NMR spectrum of ATP demonstrates well-defined α, β, and γ-phosphoryl-group resonances of ATP. Their chemical shifts depend on the state of ATP complex formation with the magnesium ion, allowing the estimation of free magnesium [15]; (**c**) scheme of the tight relationship of ATP with magnesium ion.

Magnesium, an element of cardinal physiological relevance, is the most abundant cellular divalent cation in living cells, the second most abundant intracellular cation in the human body after potassium, and the fourth most common mineral in the whole body after calcium, sodium, and potassium. Magnesium is an indispensable cofactor for the structural and catalytic actions of numerous enzymatic reactions, also acting on their substrates [5]; it is necessary for all oxidative phosphorylation reactions, energy production, protein synthesis, nucleic acid synthesis and stability, and glycolysis [6,7]. Magnesium is essential for the active transport of other ions across cell membranes; it modulates muscle contraction, normal cardiac rhythm, and neuron excitability [12,13]. Magnesium is involved in adenosine triphosphate (ATP) mitochondrial synthesis to form MgATP, which is necessary for crucial cellular reactions and signaling, including all protein phosphorylation reactions and cyclic adenosine monophosphate (cAMP) activation, which is involved in numerous biochemical cellular processes, comprising ribonucleic acid (RNA) expression, deoxyribonucleic acid (DNA) synthesis, glucose metabolism, muscular and neural cell signaling, and blood pressure control [5,7,14]. Figure 1 shows the chemical characteristics of magnesium and its close relationship with ATP.

**Figure 2:**
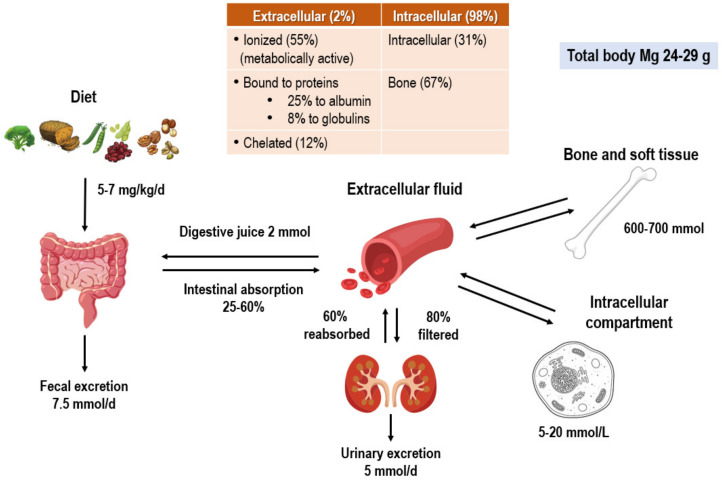
Distribution of magnesium in the body.

Magnesium is a predominantly intracellular ion (98%). Serum levels (2% of the total) are maintained within a narrow limit in healthy conditions thanks to a dynamic balance among dietary magnesium intake, intestinal absorption and excretion, renal excretion, bone storage, and the requirements of various body tissues [6,7,14] (Figure 2).

## 3. Biology of Aging

Aging is both universal and inevitable. During aging, changes occur at biological, psychological, and physiological levels. Some of these changes are benign, such as greying hair. Others result in the declining function of senses and activities of daily living and increased susceptibility to disease, frailty, and disability. In fact, advancing age is the major risk factor for numerous chronic diseases [1].

The population aged over 60 years is currently the fastest growing age group, which underscores the importance of studies on the aging process and the promotion of lifestyle choices which maximize not only longevity but, primarily, the quality of life during aging. Notwithstanding the progressive age-associated functional decline, many older adults remain physically active and with preserved cognitive functions. Even if these people are generally considered to have a favorable genetic background, accruing evidence points to a life-long healthy lifestyle as the key to successful aging.

The age-dependent decline in the physiological integrity and function of various organ systems appears to be caused by the accumulation of cellular damage leading to a progressive loss of biological function [16]. Aging occurs as a result of a series of intrinsic processes and their interactions with the external environment (e.g., ultraviolet sunlight, air pollutants and radiation, chemicals in the water, physical exercise, diet quality). Aging research has become an extensive field of study. This has come together in the description of the hallmarks of aging [9,10,11], to which a large number of researchers worldwide are dedicated and which we will discuss in detail below. Studies on the biology of aging in experimental models and in human populations have led to the emergence of theories to explain aging. The aging process is so complex that there is no single determinant that can fully explain it. Nevertheless, these studies have shown that the rate of aging can be slowed, suggesting that targeting aging mechanisms may help reduce the incidence and burden of numerous diseases and increase the healthspan (the portion of life spent in good health).

## 4. Magnesium and Aging

Chronic magnesium deficiency is frequent among older adults [8,17], which can be explained by various reasons, including a low dietary magnesium content (usual in Western diets), increased urinary excretion, or reduced intestinal absorption owing to several pathological and iatrogenic conditions [8]. Table 1 and Table 2 show the main causes of hypomagnesemia. 

Furthermore, low intracellular magnesium has been observed in older adults even in the absence of altered total serum magnesium [12]. This has been also reported in younger adults [18,19]. Mild deficits of magnesium are usually asymptomatic, and, when apparent, clinical signs are typically absent or non-specific and may be confused with common symptoms associated with aging. Table 3 shows the symptoms and signs of hypomagnesemia.

**Figure 3:**
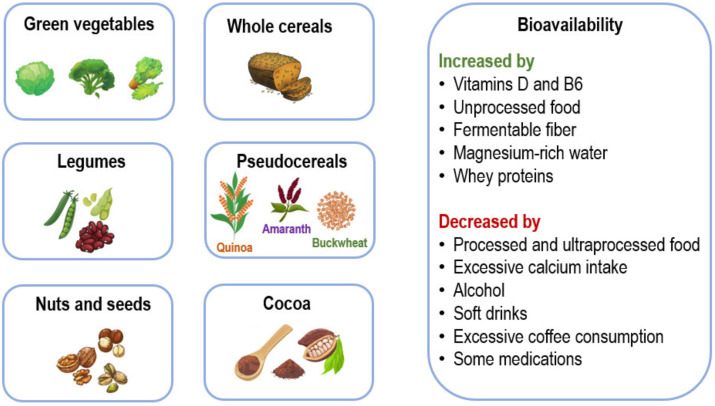
Main dietary sources of magnesium and factors that increase or decrease its bioavailability.

The high frequency of a chronic latent deficit of magnesium in older populations and its association with several diseases, medications, and surgical procedures (Table 1 and Table 2) highlights the need for recommending a diet rich in foods containing magnesium to help mitigate magnesium deficiency and its clinical consequences [8]. Figure 3 shows dietary sources of magnesium and factors that increase or decrease its bioavailability.

## 5. Magnesium Connections with the Hallmarks of Aging

**Figure 4:**
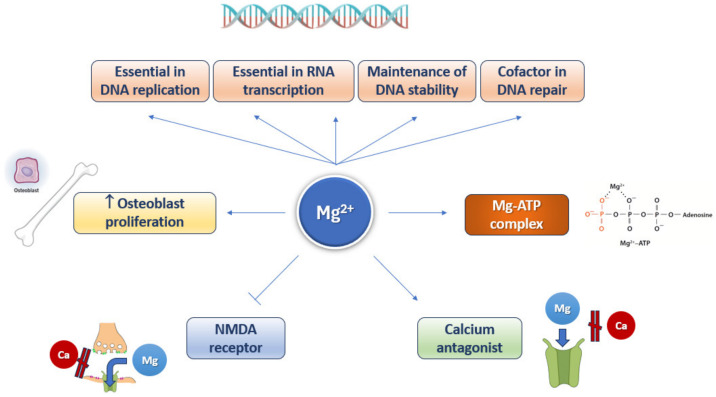
Involvement of magnesium in several cellular processes.

To the nine hallmarks of aging originally proposed in 2013 [9] others have been added, including disabled autophagy, microbiome disturbance, and inflammation [10,11]. Each of these hallmarks satisfies the following criteria: (1) it occurs during normal aging; (2) its experimental exacerbation accelerates aging; and (3) its experimental amelioration slows aging and, consequently, increases lifespan. The aging trajectory is flexible and may be modulated by dietary factors, including magnesium, and other lifestyle determinants [20]. Magnesium is involved in several cellular processes (Figure 4). 

**Figure 5:**
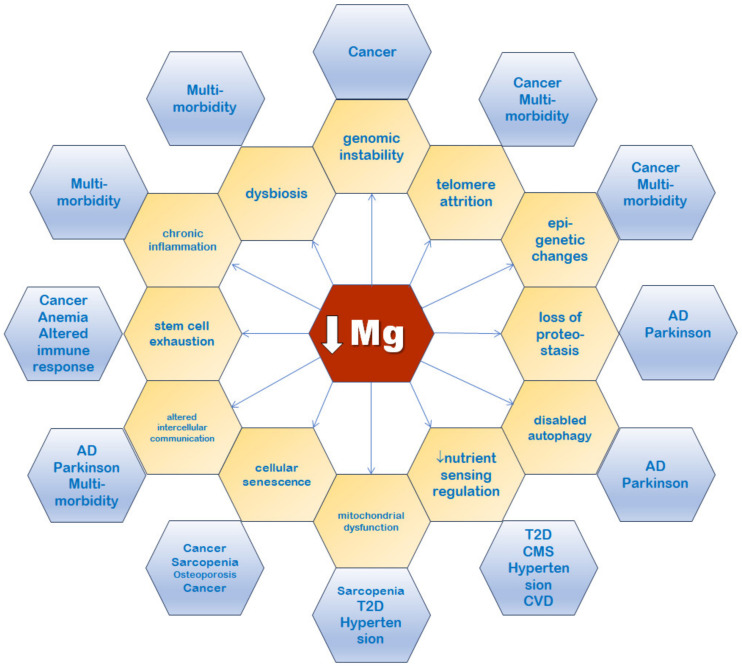
Low magnesium status is associated with all the hallmarks of aging (yellow). Examples of age-related diseases connected with the hallmarks of aging are shown in blue. AD: Alzheimer’s disease; CMS: cardiometabolic syndrome; CVD: cardiovascular disease; and T2D: type 2 diabetes.

In the next subsections, we will focus on the various hallmarks of aging, which have been closely related to magnesium (Figure 5).

### 5.1. Genomic Instability

Genomic instability refers to an increased predisposition to genomic modifications (e.g., DNA damage, mutations, and chromosomal abnormalities), and it is engendered by the effects of epigenetic alterations, oxidative stress, and deficient DNA repair and telomere maintenance [21]. DNA encodes a number of processes that detect and repair virtually all of these forms of damage; however, DNA repair mechanisms become less effective during aging, favoring the accumulation of gene mutations, which are transferred into each future copy of a cell [16,22]. Cancer is one of the results of unrepaired DNA damage or incorrect repair [23,24]. DNA mutations occur throughout life; the longer the lifespan, the more likely they are to occur. Genomic instability is arguably a chief driver of aging ultimately affecting the production of essential functional proteins needed for biochemical cellular reactions, cell-to-cell communication, and maintenance of the scaffolding [24]. 

Both minor and major DNA grooves have specific binding sites for magnesium [25]. Almost fifty years ago, magnesium was shown to be essential for the fidelity of DNA replication in DNA polymerase [26]. Magnesium contributes to stabilizing the chromatin assembly throughout the cell cycle [27], is involved in the secondary and tertiary structure of DNA, and stabilizes the conformation of DNA by hydrogen bonds or electrostatic force [28,29]. DNA fragments aggregate strongly on glass treated with magnesium in a concentration-dependent manner [30]. Thus, maintaining intracellular magnesium at a physiological level is an important determinant of DNA stability. Conversely, inadequate magnesium concentrations weaken the stability of DNA by inducing damage and oxidative stress to the double-stranded structure [31,32]. Magnesium is necessary for the activation of various enzymes involved in DNA repair pathways, such as base excision repair, nucleotide excision repair, and mismatch repair, demonstrating its crucial role in preserving genome stability [33,34,35].

### 5.2. Telomere Attrition

Telomeres are sections of repetitive nucleotide sequences (TTAGGG in humans) containing non-essential information, located at both ends of each chromosome, and protecting against its degradation and fusion with other chromosomes. This ensures that no genetic information is lost and controls the number of cell replications [36]. A small fraction of telomeric DNA (50–100 pairs of bases in human fibroblasts) is lost in each cell division due to the end replication problem, which makes telomeres gradually shorter as age advances [36,37]. When telomeres attain a critically short length, cells recognize it and turn off their replication machinery, becoming senescent, which occurs after about 50 divisions in most human cells. This limit helps to prevent cancer (limits cellular proliferative capacity) as opposed to the uncontrolled replication of cancer cells. Telomerase, which is turned off in most adult cells, can prevent telomere shortening and even restore telomere length [38]. Telomeres may undergo shortening due to oxidative stress [39,40], and shorter telomeres have been associated with increased risk of cancer, cardiovascular disease [41], and mortality, particularly at younger ages [42]. Smoking, a cardinal risk factor for cardiovascular disease, cancer, and mortality, accelerates telomere attrition [43]. Contrariwise, some but not all studies have found that physical activity protects against telomere attrition [44].

Magnesium influences telomeric chromatin structure and integrity. Over fifty percent of telomere is located in the nuclear laminae, and their laminin-binding proteins are reliant on magnesium concentrations [45]. Magnesium is also involved in telomerase regulation [46]. The catalytic component of telomerase is the telomerase reverse transcriptase (TERT); TERT exerts its biological effects via the interaction with the mammalian target of the rapamycin (mTOR) pathway, which is modulated by the concentration of magnesium [47]. Magnesium protects against age-related declining muscle regenerative potential and muscle mass loss by activating mTOR signaling [48]. Moreover, mTOR circadian fluctuations are regulated by magnesium oscillations in a MgATP-sensitive manner [49]. The expression of TERT messenger (m)RNA is modified by altered circadian rhythms accelerating the aging process [50], while magnesium fluctuations modulate the cellular clock [49].

### 5.3. Epigenetic Alterations

Epigenetics refers to modifications of genome expression without altering the DNA sequence that modulate cellular and tissue functions. Every cell contains the same DNA sequence, but the addition of epigenetic marks switches on and off the expression of certain genes as the situation demands. The epigenome can be modified by diet, other lifestyle factors, and pharmaceuticals. The complex epigenetic machinery comprises several molecules and marks, including DNA methylation, writing and erasing of these marks, histone modifications, and the proteins and enzymes which enable the reading of the marks [51,52,53]. It also includes patterns of non-coding RNA (ncRNA) expression with sizes ranging from microRNA (~22 nucleotides) to long ncRNA (>200 nucleotides) [54]. Several diseases and aging itself are associated with aberrant patterns of epigenetic marks and molecules [10,11]. The compact chromatin usually found in cells from young healthy individuals accumulates damage from multiple insults over time with aging, eventually compromising genomic integrity and cellular functioning [51,53]. As cells are exposed to environmental factors for a long period of time, epigenetic modifications may be lost, added inappropriately, or shifted around. The age-related inflammatory environment with various inhibitory molecules released from injured and stressed cells leads to accruing epigenetic alterations, ultimately changing cellular function.

Magnesium has been connected to epigenetics. Takaya et al. found that, in the offspring of magnesium-deficient pregnant rats, hepatic 11β-hydroxysteroid dehydrogenase-2 (Hsd11b2) promoters exhibited extensive hypermethylation, contributing to the down-regulation of gene expression. In this study, a low magnesium diet induced the methylation of specific cytosines in liver glucocorticoid genes generating metabolic alterations in the neonatal offsprings [55]. DNA methylation is related to gene silencing and chromatin compaction [56]. In a pilot cross-over trial of overweight healthy adults, gene-expression profiles were examined after a 4-week supplementation with magnesium, which resulted in the up-regulation of 22 genes and the down-regulation of 36 genes by ≥20% vs. placebo; many of the genes involved were linked to inflammatory pathways [57]. The results of Takaya et al. mentioned above [55] support, at least in part, potential epigenetic effects of magnesium deficiency/supplementation on inflammation. Indeed, Hsd11b1 gene deficiency, of which its promoter region is hypomethylated in rats with a calcium-deficiency [58], and the overexpression of the Hsd11b2 gene are related with improvements in metabolic features, including those linked to diabetes and hypertension [59]. Even short periods of dietary magnesium deprivation have been found to significantly downregulate telomerase and upregulate protein 53 (p53) and sphingomyelinase in cardiomyocytes, a matter which is related to important genomic changes during aging and is associated with the genesis of cardiovascular disease [60].

### 5.4. Mitochondrial Dysfunction

Mitochondria are the cellular ‘powerhouse’ producing most of the available ATP, which is the main energy source for cellular processes. They have their own genome, which is susceptible to damage, being stored in a pro-oxidation location. Mitochondria are the core for multiple signaling cascades driving the cell destiny to either survival or death by apoptosis [61]. They are the main sources of free radicals (reactive oxygen species, ROS) as a consequence of normal cellular metabolism and aerobic respiration, which, when they exceed the cellular antioxidant capacity [62], have been linked to aging and various age-associated diseases, including Alzheimer’s and Parkinson’s disease [63,64], among others. In fact, the accumulation of dysfunctional mitochondria is characteristic of aging, with decreased ATP production and increased ROS generation [10,11]. Excessive ROS damages all molecules, from proteins to DNA, causing them to mutate and, thereby, dysregulating their function. Dysfunctional mitochondria produce less ATP, reducing the energy supply and leading, over time, to chronic inflammation, oxidative stress, and cellular damage [65].

Among the many cellular functions of magnesium, the most prominent is probably its binding to ATP in the Mg–ATP complex [5,6,7]. Over a third of cellular magnesium is located in the mitochondria [66], and it is concentrated especially via mitochondrial RNA splicing 2 (Mrs2), which forms a magnesium entry protein channel and is expressed in the inner mitochondrial membrane [67]. Various tricarboxylic acid (TCA) cycle enzymes and the electron transport chain depend on magnesium, comprising 2-oxoglutarate dehydrogenase (rate-limiting step of the TCA cycle), phosphofructokinase, hexokinase, pyruvate kinase, and several subunits of the electron transport chain acting as direct activators of mitochondrial Complex V [68,69]. Disrupting magnesium mitochondrial homeostasis reduces ATP production, alters mitochondrial membrane potential, and intensifies oxidative stress [70]. Various investigations have evaluated magnesium concentration as a key regulator of mitochondrial bioenergetics, transmembrane potential, and redox state [70,71]. 

The chemical shift differences of the α- and β-phosphoryl group resonances of ATP in ^31^P-Magnetic Resonance Spectroscopy depends on magnesium complexation with ATP and gives an indirect estimate of free intracellular magnesium when combined with a simultaneous measurement of pH from the ^31^P spectrum [15]. This has allowed measurements of intracellular free magnesium in relation to hypertension and diabetes, two of the most prevalent diseases in old age [7,18,72,73] (Figure 1).

Altered mitochondrial function has been linked to magnesium deficiency by several mechanisms, comprising alterations in coupled respiration [74,75,76], suppression of the antioxidant defense system (e.g., glutathione, superoxide dismutase, catalase, vitamin E) [77,78,79,80,81], increased mitochondrial ROS production [82,83,84], attenuation of pro-survival signaling [85,86,87], triggering of calcium overload through the mitochondrial calcium uniporter [82,88,89], enhancement of mitochondrial ATP-sensitive potassium channel [90], mitochondrial permeability transition pore opening [91], and promotion of inner membrane anion channel [92]. These effects lead to the mitochondrial membrane’s potential depolarization [88]. Contrariwise, magnesium supplementation improves mitochondrial function by suppressing the overproduction of ROS [82,83], preserving the mitochondrial membrane’s potential [93,94], inhibiting the opening of mitochondrial permeability transition pore and releasing cytochrome C [95,96,97], reducing mitochondrial calcium accumulation [98,99,100], increasing anti-apoptotic B-cell lymphoma 2 family protein expression, and, concomitantly, decreasing pro-apoptotic Bcl-2-associated X protein expression [85,94], reducing apoptosis by suppressing hypoxia-inducible factor 1alpha and p38 mitogen-activated protein kinase/c-Jun N-terminal kinase signaling [94] and by downregulating autophagy [100]. 

Reduced cellular magnesium disturbs mitochondrial homeostasis also through the modulation of Mrs2, a magnesium transporter regulating magnesium flow in and out the mitochondria [70]. This significantly influences cellular energy availability and vulnerability. Experimental Mrs2 knockdown triggers the loss of electron transport chain complex I, reduces cellular and nuclear ATP levels, and makes cells more susceptible to apoptotic stimuli and oxidative stress inducers [81]. In vitro overexpression of the Solute Carrier Family 41 Member 3 (SLC41A3), identified as the first mammalian mitochondrial magnesium efflux system, was found to be linked to decreased cellular ATP [101]. Excessive activity of transient receptor potential melastatin 7 (TRPM7), another magnesium transporter, causes oxidative and nitrosative stresses [102], while oxidative stress inhibits TRPM7 current under pathological conditions associated with intracellular ATP depletion [103]. In a mouse model of progeria, a diet supplemented with magnesium increased the mitochondrial membrane’s potential and the H+-coupled mitochondrial production of nicotinamide adenine dinucleotide phosphate hydrogen (NADPH) and ATP, inducing prolonged life expectancy [104].

It has been shown that obese persons low magnesium levels are associated with oxidative stress damage via decreased antioxidant enzyme activity, lipid peroxidation, activated inflammatory pathways, and endothelial dysfunction [77]. A clear clinical manifestation of the close link between magnesium and mitochondrial function is related to muscle function. Various cross-sectional investigations have reported the strong association of hypomagnesemia or reduced magnesium intake with lessened muscle mass and function [105,106,107,108]. An intervention study among women aged over 65 years reported improvements in their physical performance scores after a 12-week magnesium supplementation vs. placebo [109]. A former study had reported decreased oxygen uptake during submaximal exercise induced by the restriction of dietary magnesium in healthy women [110]. The close link between magnesium and muscle is probably due to the mitochondrial synthesis of MgATP [8,111,112] discussed above. This link has also been defined for vascular smooth muscle [73]. 

### 5.5. Loss of Proteostasis

The homeostasis of cellular proteins is preserved by a multi-compartmental system network coordinating the synthesis, folding, disaggregation, and degradation of proteins [10,11]. Proteostasis alterations generate failed autophagy, stability loss, and accumulation of misfolded proteins [113]. Several age-related chronic diseases have been linked to the dysregulation of proteostasis, including neurodegenerative [114] and cardiovascular diseases [115]. Alzheimer’s and Parkinson’s disease have been linked to the accumulation of misfolded, unfolded, or aggregated proteins [116].

Low brain levels of magnesium have been reported in neurological disorders, e.g., epilepsy, migraine, and Alzheimer’s and Parkinson’s disease. The most accepted primary mechanisms of these pathological conditions involve the anomalous aggregation of extracellular amyloid β-protein (Aβ), tau phosphorylation, and neuroinflammation with increased expression of tumor necrosis factor-α (TNF-α) and interleukin (IL)-1β [117]. Experimental investigations found that magnesium downregulated TNF-α and IL-1β and decreased the brain accumulation of Aβ precursors [118,119]. Magnesium has also been shown to induce Aβ clearance by proteasomal degradation pathways and by decreasing the blood–brain barrier’s permeability [120]. 

A fundamental role in excitatory neurotransmission, neuro-excitotoxicity, neuroplasticity, memory, and circadian clock rhythm is played by the N-methyl-D-aspartate (NMDA) receptor [121]. Magnesium inhibits NMDA receptors, and reduced extracellular magnesium concentrations depolarize the membrane’s potential, producing increased excitability [122].

The intracellular cAMP-response element binding protein (CREB) regulates key gene expression in dopaminergic neurons. A recent study found that extracellular magnesium elevation enhanced CREB activation by NMDA receptor signaling in both rat cultured neurons and brain slices, probably independently of extracellular calcium [123].

### 5.6. Deregulated Nutrient Sensing 

The disposal soma theory states that aging results from an evolutionary trade-off among resources essential for growth, reproduction, and tissue maintenance [124]. Thus, systems sensing and interpreting cellular vital resources accessibility, i.e., energy and nutrients, or ‘nutrient-sensing systems’ are crucial for the regulation of physiological responses and processes that support growth, reproduction, and aging [124]. The development of age-related chronic diseases has been ascribed, at least in part, to modifications of various nutrient-sensing pathways, such as insulin/insulin-like growth factor-1 (IIS), 5′ AMP-activated protein kinase (AMPK), mTOR, and sirtuins [10,11,125]. The IIS pathway, fundamental for glucose homeostasis, was the first nutrient-sensing pathway to be described [126]. A downregulated IIS pathway leads to the activation of Forkhead Box O (FOXO) proteins, which have been linked to longevity by increasing insulin sensitivity [127], modulating cell cycle arrest [128], enhancing mitochondrial biogenesis, suppressing inflammation, and promoting a metabolic substrate shift from glucose to lipid oxidation [129]. These events have been associated with a decreased risk of age-related diseases, including cancer, neurodegenerative diseases, and diabetes [130]. Experimental studies in yeast, worms, flies, and mice have found that the genetic downregulation of mTOR-Complex1 (mTORC1) activity promotes healthy aging [131]. The mTOR kinase pathways sense high amino acid concentrations; AMPK and sirtuins sense nutrient scarcity, while the IIS and mTOR pathways sense nutrient abundance [124]. Upregulating AMPK and sirtuins promotes an increased lifespan via the inactivation of mTOR-C1 [132] and the stimulation of peroxisome proliferator-activated receptor gamma coactivator (PGC)-1α [133].

Accrued evidence has related magnesium deficits with modifications in insulin sensitivity and incident type 2 diabetes (T2D). Several extra- and intracellular magnesium abnormalities have been associated with T2D [12,134,135,136,137]. Low intracellular magnesium concentrations and/or low levels of ionized plasma magnesium have been detected in persons with T2D in spite of normal total serum magnesium concentrations [19,138]. Mechanisms that help explain the magnesium depletion observed in T2D include a diet poor in magnesium-containing foods (Figure 3) and increased magnesium urinary loss [139].

Two meta-analyses of prospective studies found that magnesium intake was inversely associated with incident T2D [140,141]. Likewise, in a 10-year follow-up study, hypomagnesemia was associated with glucose tolerance impairment [142]. Contrariwise, a higher magnesium intake was associated with increased insulin sensitivity [143] and with 30% decreased risk of incident T2D vs. a low magnesium intake [144,145]. Similar results were reported in the Coronary Artery Risk Development in Young Adults (CARDIA) study during a 20-year follow-up [146]. Another study found that hyperglycemia and hyperinsulinemia contributed to magnesium depletion [147], probably through excessive urinary magnesium excretion and altered magnesium transport [148]. Based on this evidence, magnesium salts supplementation has been proposed as a non-pharmacologic, safe, and inexpensive adjuvant for the metabolic control and prevention of T2D. In a systematic review and meta-analysis comprising 25 randomized controlled trials (RCTs) (12 among participants with diabetes and 6 among those at high risk of T2D), we found that magnesium supplementation reduced fasting plasma glucose in people with T2D vs. placebo and significantly improved fasting blood glucose and post 2h oral glucose load in participants at high risk of diabetes. Participants on magnesium supplementation showed improved insulin sensitivity markers [149]. In an umbrella review aiming to delineate and rate health outcomes associated with magnesium intake and supplementation, we have recently validated the association of an elevated magnesium intake with a decreased risk of T2D [150].

**Figure 6:**
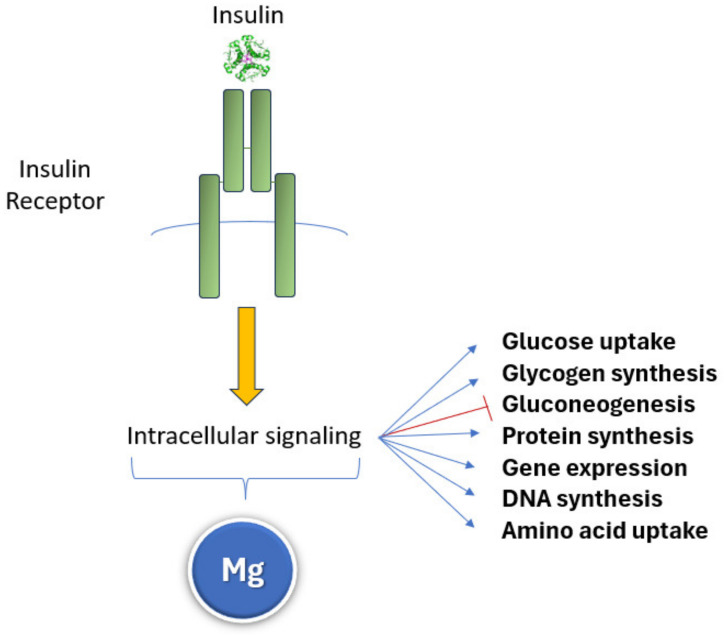
Magnesium influences all insulin signaling intracellular pathways as a cofactor of the enzymatic systems involved; hence, it modulates the effects on glucose metabolism and protein and DNA synthesis.

Low dietary magnesium has been associated with an elevated risk of metabolic syndrome, glucose intolerance, and T2D in various epidemiological studies [144,145,151]. In healthy women, a higher magnesium intake was associated with lower fasting insulin levels [152]. The total dietary magnesium intake was inversely associated with insulin responses during oral glucose tolerance tests [153]. These results are plausible because low intracellular magnesium levels disrupt the activation of all kinases in insulin signaling (Figure 6) and increase oxidative stress, generating insulin resistance and its related conditions: metabolic syndrome, glucose intolerance, and T2D [14]. 

Several metabolic disturbances have been reported in aged experimental animals, including glucose intolerance, insulin resistance, impaired oxidative phosphorylation, reduced mitochondrial biosynthesis, and decreased fatty acid oxidation [154]. Sheep undergoing magnesium deprivation exhibited impaired insulin-mediated glucose uptake [155], and magnesium supplementation postponed the onset of disease in an experimental model of diabetes [156]. Magnesium impacts metabolic pathways through its function as a cofactor of fundamental enzymatic systems in the mitochondria, where the Mg–ATP complex regulates glycolytic enzymes [68]. Magnesium is involved in the activation of mitochondrial dehydrogenases, including key rate-limiting enzymes of the TCA cycle, such as the pyruvate dehydrogenase complex [157], isocitrate dehydrogenase, and the 2-oxoglutarate dehydrogenase complex [158,159]. Furthermore, magnesium acts as a second messenger regulating insulin secretion, because it influences all insulin intracellular signaling pathways [14,160,161,162] (Figure 6). Calorie restriction (CR) has been shown to prolong lifespan and prevent age-related decline by modifying metabolic conditions in experimental models [163]. Abraham et al. found that magnesium mediated the positive effects of CR through R-loops (RNA–DNA hybrids) suppressors, while magnesium supplementation had a protective effect against the accretion of R-loops, which usually promote genomic instability and a shortening of lifespan [164]. 

### 5.7. Cellular Senescence

Senescence encompasses responses to cellular stress and damage involving irrevocable cell cycle arrest consequent to unrepairable DNA damage and senescence-associated secretory phenotype (SASP), which entails the release of inflammatory cytokines with autocrine, paracrine, and endocrine actions. Other hallmarks of aging including autophagy/mitophagy dysfunction, senescence-associated mitochondrial dysfunction, epigenetic reprogramming, and altered nutrient and stress signaling have been linked to cellular senescence [10,40], often related to the accumulation of non-telomeric DNA damage and relative biomarkers [165]. During aging, the number of senescent cells rises, increasing the risk of age-associated diseases [166]. In experimental animals, healthspan has been extended by killing senescent cells with pharmacological and genetic treatments [167]. Likewise, treatment with senolytics (comprising dietary components) reduces the number of senescent cells and postpones or prevents organism aging [168,169]. 

Some cellular alterations taking place during senescence [170] are analogous to those caused by magnesium deficit, comprising reduced protection against oxidative stress damage, culture growth, cell cycle progression, cellular viability, and increased chances for the expression of protooncogenes and transcription factors [171]. In cultures of primary human fibroblasts depleted of magnesium, Killilea et al. found that the replication capacity was lower and that senescence-associated biomarkers’ expression, such as galactosidase activity, p16INK4a, p21WAF1, and telomere attrition, was increased, in parallel with a decreased lifespan [172]. Another study in cultured endothelial cells found that short-term exposure to low magnesium media promoted features typically associated with endothelial senescence: cells became enlarged, elongated, and vacuolated, expressed senescence-associated β-galactosidase activity, and overexpressed IL-1α, considered a marker of endothelial senescence [173]. Conversely, magnesium supplementation in a mouse model of premature aging enhanced mitochondrial function, prevented tissue oxidative stress, and enhanced lifespan [104].

### 5.8. Stem Cell Exhaustion

Stem cells maintain human tissues due to their self-renewing capacity and ability to differentiate into progenitor cells, generating cellular diversity of various tissues [174]. Their key functions deteriorate due to extrinsic and intrinsic factors promoting aging and age-related diseases. For example, age-related reduction in the hemopoietic cells’ regenerative potential may result in the diminished production of adaptive immune cells (immunosenescence), which is associated with an increased risk of anemia and myeloid malignancies [175]. Immunosenescence is often manifested as the subclinical accumulation of pro-inflammatory factors and ‘inflammaging’ [176].

Older adults present features of immunosenescence with variable severity, including decreased total number and proliferative capacity of hematopoietic stem cells. The presence and severity of frailty is linked to the severity of immunocompetence [177]. Overall, older people have a mild degree of immunosuppression due to immunosenescence, added to age-associated multimorbidity, organ decline, malnutrition, functional failure, frailty, geriatric syndromes, and polypharmacotherapy [177,178]. The combination of all these factors may help to explain the increased susceptibility to infections with a worse prognosis, including COVID-19 [179], to the development of neoplasms and autoimmunity, and to a reduced ability to heal cutaneous lesions in old age.

Several investigations support the role of magnesium in the immune response. Magnesium is a cofactor for the synthesis of immunoglobulins (Ig), immune cell adherence, C3 convertase, IgM lymphocyte binding, antibody-dependent cytolysis, T helper–B cell adherence, and macrophage response to lymphokines [180,181]. Magnesium reduces the expression and secretion of proinflammatory molecules, such as substance P, by controlling the activity of nuclear factor kappa-light-chain-enhancer of activated B cells (NF-kB) in normal magnesium conditions and triggers an increased activation of NF-kB and cytokine production in suboptimal magnesium concentrations [182]. Magnesium also modifies acquired immunity through the regulation of lymphocytes’ development and proliferation [183]. Most of this research has been carried out in experimental animals fed diets poor in magnesium. These animals exhibited an increased number and altered function of polymorphonuclear cells, which were associated with increased phagocytosis [184]. Mast cell proliferation and function is also disrupted by magnesium deficit and could be involved in liver fibrosis and steatosis [185,186]. Human Fas-induced B cell apoptosis is a magnesium-dependent process [187]. Other studies confirm that magnesium-deficient experimental animals display decreased specific immune responses, exacerbated immune stress responses, and increased inflammation [184,188,189,190]. 

Interestingly, the fundamental significance of magnesium in immunity was unveiled after the characterization of XMEN (X-linked immunodeficiency with magnesium defect, Epstein–Barr virus infection, and neoplasia), a primary immunodeficiency due to a genetic deficiency of the transporter MAGT1 [191,192,193], which suggested that magnesium might function as a second messenger in cellular immune signaling.

Even if magnesium has a crucial role in cellular replication as well as in DNA and RNA synthesis, the influence of altered intra- and extracellular magnesium homeostasis on the hematopoietic tissue is not extensively studied. Bone mesenchymal stem cells (MSCs) may differentiate into different strains, e.g., osteoblasts and adipocytes [194]. Adipocytes are negative regulators of hematopoiesis, while osteoblasts are promoters of it [195,196]. High magnesium concentrations have been associated with increased osteoblastogenesis; thus, magnesium deficiency, at least in part, seems to be detrimental to bone health [197,198].

Magnesium deficiency increases the transcription of multipotency markers and tissue-specific transcription factors in human adipose-derived MSCs exposed to a mixture of natural molecules, i.e., hyaluronic, butyric and retinoid acids, which tune differentiation. Thus, magnesium deprivation may generate stressful conditions that modulate stem cell plasticity and differentiation potential [199].

### 5.9. Altered Intercellular Communication

The communication between cells is essential for coordinating cell functioning at all levels (organs, tissues, whole body) and encompasses soluble factors including cytokines, chemokines, neurotransmitters, and growth factors, recognized by specific receptors at the cell surface [200]. Intercellular gap junctions contribute to intracellular signaling by exchanges of ions and regulatory molecules, which are crucial for cell proliferation, differentiation, and apoptosis. Aging is associated with significant alterations in cell-to-cell communication via neuronal, endocrine, and neuroendocrine routes [9]. Inflammation is one of the most extensively studied and relevant intercellular communication events and it is modified with aging. As mentioned, a significant feature of aging is a prevalent chronic, low-grade, systemic inflammation—inflammaging [201]—which is linked to and predictive of frailty [202], T2D [203], neurodegenerative diseases [204], and mortality risk [205,206]. Inflammaging may have several causes, including increased production of free radicals, increased secretion of pro-inflammatory cytokines and adipokines, enhanced activation of the NF-κB pathway, and modifications in the gut microbiome and intestinal permeability [207,208,209]. In the original proposal, inflammation was included as part of the hallmark ‘altered intercellular communication’ [9]; nevertheless, it is now considered as another of the hallmarks of aging owing to its large involvement in the aging process and its role underlying other hallmarks such as cellular senescence, stem cell exhaustion, and the recently proposed altered gut microbiota [210,211], (see Section 5.11).

Magnesium and its transport systems are key modulators of various intracellular signaling pathways’ communication. The NMDA receptor takes part in excitatory neurotransmission, neuro-excitotoxicity, neuroplasticity, memory, and circadian clock rhythm, all of which are modified by age [121]. Magnesium blocks the ion channel of the NMDA receptor and prevents its excessive activation. A reduction in extracellular magnesium depolarizes the membrane potential, leading to hyperexcitability [122]. The magnesium transporter TRPM7 acts as a kinase; various kinase substrates have been defined, including myosin IIA heavy chain, annexin-1, and calpain, validating the role of TRPM7 in multiple cellular functions, comprising vascular dilation and contraction, growth, apoptosis, migration, anti-inflammatory responses, and cell adhesion [212,213,214]. Magnesium influx through TRPM7 contributes to the activation of the phosphoinositide 3-kinase (PI3K)/Akt/mTOR signaling pathway in tumor B-lymphocytes and produces quiescent/proliferative metabolic transitions. As TRPM7 is widely expressed, it has been suggested that TRPM7 may impact metabolic transitions related to rapid cellular proliferation and malignancy in many tissues [70,215]. Furthermore, the overexpression of SLC41A1, a Na+/Magnesium exchanger which allows magnesium efflux, remarkably attenuated the phosphorylation of kinases involved in anti-apoptotic and, hence, pro-survival cellular events [87].

### 5.10. Compromised Autophagy

Alterations in autophagy are observed in numerous aging conditions including immunosenescence and neurodegeneration [216,217]. The prominent activation of autophagy has been shown to increase mouse lifespan [218]. In addition, autophagy has been linked to improvements in immune response to vaccination in older humans by overcoming immunosenescence [219]. In the original proposal of the hallmarks of aging, autophagy was considered together with the hallmark ‘altered proteostasis’. The updated proposal includes autophagy as another hallmark owing to its relevant role in regulating other hallmarks, such as DNA repair and nutrient sensing/metabolism [220]. 

Magnesium is essential for chief cellular processes, comprising energy metabolism, proliferation, and apoptosis [8,221]. Some mechanisms by which magnesium modulates cell proliferation include cell cycle inhibitors p53 and p27 and other negative cellular proliferation modulators (e.g., Jumonji and numblike) [221,222]. Nevertheless, the evidence for magnesium’s role in cellular apoptosis remains uncertain. In some experimental models, deprivation of magnesium triggered cell death by apoptosis, while restriction in dietary magnesium accelerated apoptosis [221,223,224]. However, intracellular levels of free magnesium were increased in cells undergoing apoptosis, constituting an initial event during the apoptosis process [225]. Endonucleases that depend on calcium and magnesium have been involved in DNA breaks during apoptosis [226]. Magnesium may perform as a ‘second messenger’ for the downstream phases in apoptosis [187]. A lack of magnesium increases the tendency to oxidative damage [14,81], enabling alterations in membrane integrity and function.

### 5.11. Dysbiosis

In the past few decades, advances in research on the gut microbiota/microbiome have put in evidence its importance in human health. Recent developments in next-generation sequencing methods have consented researchers to investigate modifications in the microbiome occurring during aging [227] and, perhaps, will help to elucidate their influence on the host’s physiology. According to recent investigations, gut microbiomes turn out to be increasingly unique in each individual during aging, a process which starts in mid-to-late adulthood. Wilmanski et al. evaluated three independent cohorts including >9000 participants and found that the uniqueness in microbiome composition was strongly and positively associated with known metabolic markers of the microbiome implicated in inflammation, immune regulation, aging, and longevity. Healthy participants aged over 80 years exhibited a sustained microbial shift towards a unique composition that was absent in less healthy ones. In participants aged > 85 years, maintaining a high relative abundance of *Bacteroides* and a low gut microbiome uniqueness were associated with significantly reduced survival during a 4-year follow-up [227]. Modifications in microbial populations and the cross-talk along the gut–brain axis regulate inflammatory nociception, inflammatory responses, and immune homeostasis [228], which together with the age-associated loss of structural integrity of the gut and other barriers (e.g., blood–brain barrier) can provide key advances in age-related inflammation-associated diseases.

Although so far there are few studies exploring the relationship between magnesium and microbiota composition, some research suggests that the gut microbiota could be an intermediary, linking magnesium intake to some age-related conditions. In experimental models of rheumatoid arthritis (RA), Laragione et al. showed that a high-magnesium diet significantly reduced the expression of IL-6, IL-1β, and TNFα in parallel with decreased joint damage and arthritis severity. In addition, animals receiving a high-magnesium diet had higher numbers of IL-10-producing T cells and Foxp3+ regulatory T (Treg) cells. Fecal material transplantation from mice on the high-magnesium diet reproduced the phenotypes observed in the diet-treated mice (reduced arthritis severity and modified immune profiles). Analyses of the intestinal microbiome with 16S rDNA sequencing showed diet-specific modifications, comprising reduced levels of RA-associated *Prevotella* in the high-magnesium diet group and increased levels of *Bacteroides* and other bacteria associated with increased production of short-chain fatty acids. [229]. Future similar studies could be carried out on other autoimmune and inflammatory diseases.

Hypomagnesemia is a commonly reported side effect of proton pump inhibitors (PPIs), a first-line treatment for gastric acid-related disorders. The gut microbiome may contribute to the development of PPI-induced hypomagnesemia, as PPI use affects the composition of the gut microbiome via the increase in the luminal pH, altering magnesium intestinal absorption [230]. A study in an experimental model found that methotrexate-induced intestinal and liver injuries were significantly alleviated using a magnesium isoglycyrrhizinate treatment, which also reshaped the gut microbial composition and inhibited bacterial translocation into the liver [231]. Del Chierico et al. evaluated whether magnesium supplementation’s positive effects could be mediated by the gut microbiota in a murine model of colitis. Dietary magnesium supplementation increased the population of bacteria linked to intestinal health and metabolic homeostasis and reduced bacteria associated with inflammation and related human diseases, such as inflammatory bowel disease [232]. An experimental study in weaned pigs showed that dietary supplementation with potassium–magnesium reduced inflammatory cytokines in the jejunal mucosa, a phenomenon which was partially related to changes in colonic microbiota composition [233].

Gut microbiota, as well as magnesium, have been linked to mental conditions, including depression and anxiety disorders. Three experimental studies have shown that magnesium has a significantly positive impact on intestinal microbiota and mental manifestations. In one study, mice on a low magnesium diet for 6 weeks experienced a significant change in their gut microbiota and increased anxiety-like behavior [234]. In another study, the same researchers reported that mice exposed to a magnesium-deficient diet for 6 weeks experienced increased depressive-like behavior and alterations in gut microbiota [235] and demonstrated a relationship between the change in gut microbiota and change in depressive behaviors. In a third study [236], Pachikian et al. showed that mice fed a magnesium-deficient diet for up to 21 days had reduced concentrations of bifidobacteria independently of any other changes in nutrient intake vs. mice that had been fed a magnesium-repleted diet. Mice fed a low-magnesium diet had also a lower mRNA content of factors controlling the gut barrier function in the ileum (zonula occludens-1, occludin, proglucagon) and a 2-fold higher mRNA content in the liver and/or intestine of TNF-α, IL-6, CCAAT/enhancer binding protein homologous protein, and activating transcription factor 4, reflecting inflammatory and cellular stress.

Even if the evidence so far comes mainly from animal studies, the findings are interesting and relevant. While human studies are available, it is still wise and necessary to avoid magnesium deficit by promoting the consumption of foods with a high magnesium content (Figure 3). 

### 5.12. Inflammation

We have already mentioned that age-related chronic low-grade inflammation, or inflammaging, is involved in a wide range of chronic diseases [8,203]. Aging correlates with high blood levels of inflammatory mediators, such as IL-1, IL-6, C-reactive protein (CRP), interferon (IFN)α, and several others [237]. It is due to its paramount importance mediating numerous conditions and altered pathways that, in the updated proposal of the hallmarks of aging, inflammation was included as an independent one [10,11].

In vitro studies have shown that low concentrations of magnesium initiate an excessive production and release of IL-1β and TNF-α, activate phagocytic cells, open calcium channels, activate the NMDA receptor and NF-κB signaling, and stimulate the synthesis of nitric oxide and other inflammatory markers [238,239,240,241]. Increased ROS production and altered calcium homeostasis triggered by low magnesium levels have been shown to further impair mitochondrial function [238], increasing the release of inflammatory markers. In addition, magnesium deficit enhances platelet adhesiveness and aggregation and inhibits endothelial cells’ migration and growth, potentially disrupting microvascular structure and function [240,242]. Adequate magnesium concentrations inhibit ROS production and mast cell degranulation, protect epithelial cells, and lessen the oxidative and inflammatory damage to cells and blood vessels [243]. Magnesium concentration is reduced in acutely inflamed tissues by the activation of the IL-33/ST2 axis, confirming the importance of magnesium in inflammatory pathways [244].

**Figure 7:**
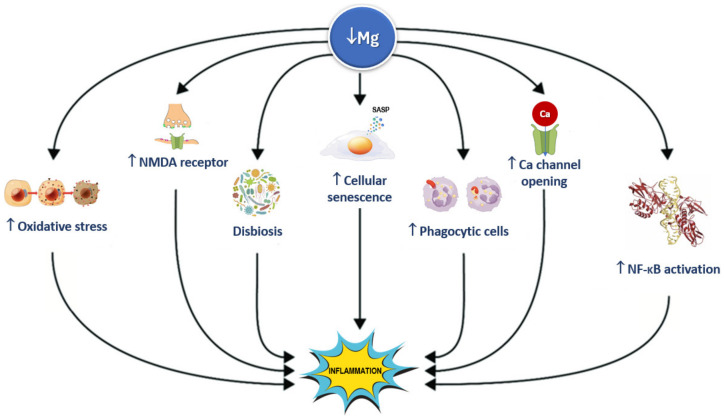
Magnesium deficiency induces inflammation through several signaling pathways. NF-kB: nuclear factor kappa-light-chain-enhancer of activated B cells; NMDA: N-methyl-D-aspartate; and SASP: senescence-associated secretory phenotype.

In experimental models, magnesium deprivation led to increased inflammatory parameters including TNF-α, IL-6, IL-1-β, plasminogen activator inhibitor-1, and vascular cell adhesion molecules [188]; increased liver production and secretion of acute phase proteins (i.e., fibrinogen, α2-macroblobulin, complement) [184,240]; and increased the number of circulating inflammatory cells [181,239]. Low magnesium levels have been also related to endothelial dysfunction, which has been linked to the release of inflammatory mediators as well [242]. Contrariwise, supplementation with magnesium sulphate was found to facilitate anti-inflammatory effects in murine macrophages through the reduced upregulation of inflammatory mediators induced by endotoxin and NF-κB, along with the inhibition of L-type ion channels and the activation of PI3K [245]. Magnesium has calcium channel-blocking effects that lead to the downstream suppression of IL-6, NF-κB, and CRP [246]. Figure 7 illustrates several signaling pathways by which magnesium deficiency induces inflammation.

In epidemiological studies, low serum magnesium concentrations and a low dietary magnesium intake were associated with low-grade systemic inflammation [247,248,249]. Further studies have confirmed an inverse association of magnesium intake and serum magnesium with inflammatory markers [250,251,252]. One of the most relevant contributions comes from the Nurses’ Health Study, in which magnesium intake was inversely associated with serum CRP concentrations [248] and related to the presence of metabolic syndrome [250]. Likewise, King et al. using data from 70% of the 1999–2002 National Health and Nutrition Examination Survey (NHANES) population not taking supplements found that magnesium intakes below the RDA were significantly associated with elevated CRP [247]. Results from a large Finnish cohort corroborated again the inverse association of a low dietary magnesium intake with serum CRP levels [252]. 

A former meta-analysis including eight RCTs showed that, after magnesium supplementation, serum CRP concentrations were reduced, independently of the duration and dosage of magnesium supplementation [253]. We have recently performed an updated meta-analysis including 17 RCTs and found that magnesium supplementation significantly reduced different human inflammatory markers, specifically nitric oxide and CRP concentrations [254].

## 6. Concluding Remarks

Magnesium is an essential cation in the human body because it regulates numerous physiological and pathological processes. Magnesium deficiency is very common in older adults. Chronic low-grade inflammation (inflammaging) is recurrently associated with a number of chronic diseases linked to aging and with the aging process itself. Because magnesium inadequacy has been associated with an excessive production of inflammatory markers and ROS, we have formerly hypothesized that a chronic magnesium insufficiency may be one of the intermediaries of the relation between inflammaging and age-related diseases. As shown in this review, there is evidence that magnesium is related to all the hallmarks of aging (Figure 5). An optimal magnesium balance during one’s life course may help preventing inflammaging and its related consequences. However, while it is prudent to keep an adequate magnesium balance through a diet with sufficient magnesium-rich foods (Figure 3) and/or supplements when indicated, it is crucial not to lose sight of the fact that the aging process is complex and that correcting just one of the multiple determinants may not have any effect in the long run. However, the present review intended to clarify possible mechanisms that can explain the close relationship of magnesium with the hallmarks of aging, without claiming that this cation is the only or most important determinant of this multivariate process. 

How does one clinically approach magnesium in old age? First, it is essential to ensure that seniors have a diet that is adequate in its components and includes foods rich in magnesium (Figure 3). If, for any reason, the person has difficulties in following this type of diet, supplementation with magnesium salts should be considered, especially in patients affected by diseases or conditions which are more frequently associated with hypomagnesemia (Table 1 and Table 2). In these cases, it is advisable to measure the patient’s serum magnesium concentration, which is the only widely available clinical method, knowing that levels below the normal range will reveal only severe magnesium depletion because the amount in the serum is much lower than the intracellular magnesium content (Figure 1). For this reason, values that tend to be low, even those referred to as normal by the laboratory, should be considered inadequate. It is also necessary to consider polypharmacy; hence, an adequate intake of foods rich in magnesium should be recommended in the first place, considering supplementation only for people who cannot follow such a diet. It is important to consider that magnesium supplementation may cause diarrhea when given in large doses; therefore, adequate dose titration is necessary. In addition, assessment of renal function before supplementation is important, while magnesium supplementation in patients with renal failure should be undertaken cautiously.

Significant progress has occurred in aging research in the past decades, but a unified theory of aging that can fully explain such a complex process is understandably still missing. The study of the hallmarks of aging as isolated events may help researchers better define the mechanisms underlying the complex aging process. However, these mechanisms can interact with each other. For example, the pro-inflammatory state results from mitochondrial dysfunction and cellular senescence, among others (Figure 7). The possible synergistic effects of these interactions are not simple research questions to address. However, a factor with multiple effects on the different hallmarks of aging such as magnesium can help us understand the close links among them and possibly allow us to suggest that such interactions have strengthening effects on one another.

The possibility that maintaining a suitable magnesium balance over the course of a lifetime may become an inexpensive and safe strategy contributing to healthy aging is an intriguing hypothesis, which needs to be further explored by future well-designed studies.

## Figures and Tables

**Table 1:** Main pathological causes of hypomagnesemia.

**Genetic disorders of renal magnesium wasting** • Bartter’s syndrome • Gitelman’s syndrome • Familial hypomagnesemia • Hypomagnesemia with secondary hypocalcemia • Isolated dominant hypomagnesemia **Malabsorption diseases** • Cystic fibrosis • Chronic pancreatitis • Celiac disease • Whipple disease **Diseases that can be associated with hypomagnesemia** • Diabetes • Hypertension • Malnutrition (inadequate intake) • Alcohol abuse • Chronic diarrhea • Hyperaldosteronism • Hyperthyroidism • Hypoparathyroidism

**Table 2:** Main iatrogenic causes of hypomagnesemia.

**Medications** • Chronic use of diuretics • Chronic use of proton pump inhibitors • Use of nephrotoxin drugs (e.g., amphotericin B, cisplatin, cyclosporine, aminoglycosides) **Surgery** • Bariatric surgery (gastric bypass, sleeve gastrectomy, biliopancreatic diversion) • Oncologic surgery for gastrointestinal tumors • Parathyroidectomy

**Table 3:** Symptoms and signs of hypomagnesemia.

**Neuromuscular and central nervous system** • Muscle cramps • Muscle weakness, fasciculations, tremors • Lethargy, tetany • Vertigo, nystagmus • Depression, psychosis • Carpopedal spasm • Convulsions • Athetosis, chorea-like movements **Cardiovascular** • Atrial tachycardia, fibrillation • Supraventricular arrhythmias • Ventricular arrhythmias • Torsade de pointes • Digoxin sensitivity **Electrolyte disturbance** • Hypokalemia • Hypocalcemia **Complications of magnesium deficiency** • Alterations in glucose homeostasis • Hypertension • Atherosclerotic vascular disease • Myocardial infarction • Osteoporosis **Other disorders** • Asthma • Migraine • Chronic fatigue syndrome • Impaired athletic performance

## Data Availability

Not applicable.
